# E-health roadmap for COVID-19 vaccine coverage in Iran

**DOI:** 10.1186/s12889-021-11419-y

**Published:** 2021-07-23

**Authors:** Elham Maserat, Leila Keikha, Somayeh Davoodi, Zeinab Mohammadzadeh

**Affiliations:** 1 Department of Medical Informatics, Faculty of Medical Sciences, Tarbiat Modares University, Tehran, Iran; 2 Health Information Management, Department of Medical Library and Information Sciences School of Allied Medical Sciences, Zahedan University of Medical Sciences, Zahedan, Iran; 3 Department of Health Information Management, School of Paramedicine, Hormozgan University of Medical Sciences, Bandar Abbas, Iran; 4 Department of Health Information Technology, School of Management and Medical Informatics, Tabriz University of Medical Sciences, Tabriz, Iran

**Keywords:** COVID-19, Vaccine, Roadmap, E-health

## Abstract

**Background:**

Vaccination is the effective and long-term pharmacological solution to deal with COVID-19. Information technology (IT) and electronic immunization can be effective in accelerating and improving vaccine coverage. The aim of this paper is to develop multi-dimensional framework of e-health roadmap to response Covod-19 pandemic and examine the role of IT for improving vaccine distribution in Iran.

**Methods:**

The study methodology was based on a two-stage Delphi method which included literature studies at the beginning. Key steps in creating a roadmap in this study include definition, development and evaluation. The initial conceptual model was developed after literature review. Proposed roadmap was reviewed and evaluated in two stages based on the Delphi method by experts in the fields of E-health.

**Results:**

In the e-health roadmap model, 14 stages of vaccine distribution were presented in three phases of vaccination and then were determined the type of technology in each phase. The 4 conceptual models were approved based on the two stages Delphi approach in a survey of 14 e-health experts. In the second phase of the Delphi process, the selected items were sent back to the specialists to verification. Then e-health roadmap was confirmed by experts and was finalized the approved model.

**Conclusions:**

The technology-based roadmap is one plan in the form of a transfer strategy that aligns goals with specific technical solutions and helps to meet them. This roadmap empowers decision makers to decide on alternative paths and achieve goals.

## Background

Since the outbreak of COIVD-19 disease in December 2019 [1, 2], various strategies have been proposed to control this disease, including isolating suspected individuals, closely monitoring contacts, collecting epidemiological and clinical data from patients, and developing diagnostic and therapeutic methods [3, 4].

In the meantime, a lot of research has been done in the field of facilitating and accelerating the diagnosis of the disease, creating vaccines and possible treatments, and also understanding the socio-economic effects of this disease as well [5]. Some pharmacological and non-pharmacological measures are taken to control the disease. Currently in most countries, the main strategies to prevent the spread of the virus is mask wearing, hand washing and limiting human-to-human transmission [6, 7]. But the effectiveness of these methods is short and have psychological, economic and social impact to people.

Iran is one of the countries with the highest morbidity and mortality of COIVD-19 [8]. and there is 2,237,000 affected cases and 66,732 deaths in Iran [9] The first confirmed case of coronavirus infection observed in Iran on 19 February 2020. Then the virus spread rapidly throughout the country and affected many people [10]. Iran is one of the countries with the highest rate of corona virus with 2,237,000 affected cases and 66,732 deaths [9]. The outbreak of this disease had many challenges for the country. The strategies were adopted to control it similar to other countries in the world [8]. And lots of measures were taken in Iran to control the epidemic, including translation of WHO guidelines for COVID-19 prevention and treatment [11].

One of the effective and long-term pharmacological solutions to deal with epidemics such as COVID-19 is an immunization that should be done globally [12, 13]. Vaccination programs will be desirable if done in a timely manner and with adequate acceptance and coverage [14]. This method has reduced mortality from other similar infectious disease such as smallpox, polio, rabies, typhoid and plague [15]. Vaccination strengthening the level of immunization in individuals and leads to clinical and socioeconomic benefits and herd immunity [13, 16].

Despite of the effectiveness of vaccination, there are some challenges associated with adequate and complete vaccination coverage to achieve immunity or prevention. A logical principle is that if the vaccination coverage is not complete, immunity will not occur [17]. To addressing the challenges and try to success of vaccination programs, many experts recommend an interdisciplinary approach with the aid of technology solutions [15].

New technologies are critical to the advancement and success of medical science [18]. The use of health information technology is essential to improve the quality of care delivery and reduce health care costs, especially in the management of chronic diseases [19]. Studies confirmed the effect of information technology and electronic immunization in accelerating and improving vaccination coverage [20].

Technological innovations lead to timely access to information and facilitate communication. However, the vaccination schedule is complex, but Information technology can be used in various areas of COIVD-19 management, such as prevention, screening, diagnosis, treatment and follow-up [10, 21, 22]. Because of its prevalence, influence and flexibility, technology provides promising tools to overcome vaccination barriers in all levels including families, health care providers and the broader community.

In addition to adequate vaccines, there must be an adequate community acceptance of the vaccine. With training and providing proper information through communication technologies, the negative attitude towards the vaccine can be reduced and its acceptance rate can be increased [13, 23].

The complete and effective vaccine for SARS-CoV-2 will require the collaboration of all levels of society [24]. The purpose of this paper is to develop the multi-dimensional framework to map existing interventions and areas in which technology for improving vaccine communication in Iran. With this roadmap, we can review IT capabilities and barriers of vaccine communication at several levels, including policy makers, Health care providers and people.

## Method

### Research methodology

The study methodology for the development of the proposed framework was based on a two-stage Delphi method which included literature studies at the beginning. Key steps in creating a roadmap in this study include definition, development and evaluation. The Delphi method is used to gather expert opinions about complex components. This method collects, synthesizes, and reports experts diverse responses (both qualitatively and quantitatively), and do all this in an effective, timely and objective approach [25].

**Fig. 1:**
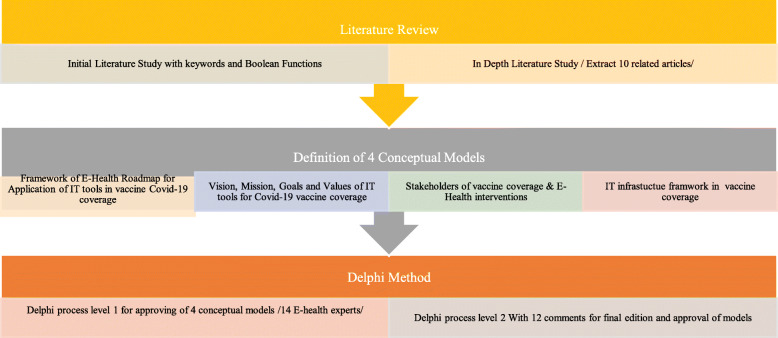
Research Methodology flow

In this study, a comprehensive review was conducted with the aim of extracting the components of the models and e-health roadmap. In this review, related articles were extracted based on search strategies and reviewed by experts in this field. The 8 related articles were extracted and presented in Table 1. Based on the literature review, the models were defined, reviewed and approved by experts in two Delphi stages (Fig. 1).

**Table 1:** Result of the studies in the field of e-health interventions and vaccination coverage

No	Aims of interventions	IT Tools For Interventions	phases	Intervention types and processes	Monitoring & evaluation
1	Improve childhood immunization [26]	- Electronic Health Record (EHR) - Barcode technology	After and during vaccination	- EHR alert and reminder - Rapid and accurate input of information via scanning of the vaccine barcode.	- Improving the quality of care - Improve communication between health care providers - Reduce missed immunization - Tracking and reporting vaccination program
2	Improve vaccine coverage [27]	SMS reminders	pre vaccination	Alert for track individual	Improve immunization coverage and timeliness
3	Inform and improve decision-making for polio programming and response [28].	GPS, smartphone	During vaccination	Electronic questionnaire upload to smartphone and GPS co-ordinate for each household	- Enabling collect standardized data in real-time - Help stakeholder to decision-making, - Save time and cost
4	Improve immunization Coverage and timeliness [29].	SMS reminder	Pre vaccination	2 SMS reminders—3 days and 1 day before measles immunization	Improve measles vaccination coverage
5	Improve the coverage and quality of the vaccination [30]	GIS	After vaccination	Create map for identifying vaccination state for each lot and for different type of vaccine	Increase the coverage and quality of the vaccination services.
6	Increase influenza vaccination rates [31]	Text and e-mail message	Before vaccination	Protect children who do not receive vaccination	Cost effective improve the rate of vaccination
7	Increase their vaccination coverage [32]	Website	After vaccination	Educational program through website to increase knowledge	Possibility of increasing vaccination coverage
8	Improving completion of the infant primary immunization series [33]	SMS reminder	Pre vaccination	Three SMS reminders one week before the second and third dose.	SMS reminders would be helpful for remembering appointment
9	- Assess the coverage of vaccination - Introduce Global Positioning System (GPS) and Google Earth as new tools for vaccine coverage [34]	GPS	After vaccination	Spatial mapping using a GPS receiver and Google Earth	- Immunization coverage identified - Probable reason found for partial/non-immunized children.
10	Timely creation of an electronic registry [35]	mHealth	Pre vaccination	Pre-registered people with card and during vaccination scan the card	Identify who had not yet been vaccinated

#### Literature review processes

The literature was reviewed in two initial and in-depth stages. These two steps include the following:

### First step: initial literature study

Initially, the search was performed in the Cochrane Library, Scopus, Web of Science, ProQuest, PubMed and Google Scholar using the eligible keywords including Vaccination AND (medical informatics OR Telephone OR telemedicine OR social media OR internet OR Telemedicine OR Geographic Information Systems) AND coverage.. Then articles that used various health information technology tools in vaccine communication and coverage as an intervention were extracted based on title and abstract. The websites of leading organizations in this field, such as the World Health Organization (WHO) and the Centers for Disease Control and Prevention (CDC), were also examined and related information was collected.

For example, the search strategy in the PubMed database was executed as follows:

(COVID-19[title/abstract] OR COVID-19[title/abstract] OR Coronavirus[title/abstract] OR Novel Coronavirus [title/abstract] OR 2019-nCoV[title/abstract] OR SARS-CoV- 2[title/abstract] OR SARS2 [title/abstract]) AND (Information technology[title/abstract] OR Health information technology[title/abstract] OR Telehealth[title/abstract] OR Tele-health[title/abstract] OR Telecare[title/abstract] OR Mobile health[title/abstract] OR mHealth[title/abstract] OR Electronic health[title/abstract] OR eHealth[title/abstract]) OR Registry[title/abstract]) AND (Vaccine[title/abstract] OR Vaccination[title/abstract] OR vaccine coverage[title/abstract] OR vaccination coverage[title/abstract] OR vaccine communication[title/abstract] OR vaccination communication [title/abstract]) AND (Roadmap[title/abstract])

In the selection process of articles, firstly different databases including pubmed, scopus, and google scholar were searched by defined keyword“ COVID-19, Coronavirus, Novel Coronavirus, 2019-nCoV, SARS-CoV- 2, Information technology, Health information technology, Telehealth, Tele-health, Telecare, Tele-health, Mobile health, mHealth, Electronic health, eHealth, Registry, Vaccination, vaccine coverage, vaccine communication, vaccination communication, Roadmap “76 papers were extracted from PubMed and 6 article from Scopus. The first 10 page of google scholar were also reviewed for eligible articles. Then extracted articles were screened based on mentioned criteria by two researchers and finally 10 articles were included in this study.

After literature review, the 4 conceptual models were drawn in multiple aspects. This 4 models including application of IT tools in three phase of COVID-19 vaccination, vision, mission, goals and values of IT tools for COVID-19 vaccination coverage, IT infrastructure framework in vaccine coverage and stakeholders of vaccine coverage & E-Health interventions.

### Second step: in depth literature study

Based on the results of the initial literature review, the necessary steps for conceptual models related to the distribution of vaccine were formulated and designed. After reviewing the full text of the articles, irrelevant and less relevant articles were removed. Then articles in which information technology tools were used in the field of vaccine distribution, were examined in more depth. The detail information of 8 articles based on search strategy including the objectives of each intervention, IT Tools for Intervention, Intervention type and process, and performed Monitoring & evaluation were extracted in Table 1.

#### Delphi processes

In this phase, the proposed roadmap model was approved by experts during the Delphi processes. Proposed roadmap was reviewed and evaluated in two stages based on the Delphi method by experts in the fields of E-health. In the first stage, proposed roadmap and the conceptual models were sent to 14 experts from Tehran, Tabriz, Zahedan, Hormozgan, Mazandaran and Esfahan Universities of Medical Sciences. The expertise and jobs of the professionals participating in the Delphi stages are as follows. All 14 experts had a specialized doctorate degree and all of them are working in medical universities and in departments that are completely related to the objectives of this study. Among these experts, 9 were university faculty members specializing in health information management, 3 were medical faculty members specializing in medical informatics, 1 was a software specialist and 1 was a medical librarian. The researchers explained the purpose of the study to the participants and maintained confidentiality of their responses. Then the Expert opinions were collected and examined. According to the opinions of experts in the first stage of the Delphi process, after raising issues in several meetings of the research team, the necessary corrections were applied to proposed roadmap and conceptual maps. In the second phase of the Delphi process, the corrected items were sent back to the specialists. At this stage, the roadmap was approved by experts and the approved model was finalized.

## Results

In total, four models were defined including, framework of E-health roadmap for application of IT tools in COVID-19 vaccination coverage, perspectives of IT tools for COVID-19 vaccine coverage, IT infrastructure framework in vaccine coverage and stakeholders of vaccination coverage & E-health interventions.

### Review the selected papers in the field of coverage vaccination based on IT technologies

In this study, all relevant scientific sources in the field of e-health interventions and vaccination coverage were reviewed. According to the information obtained from mentioned articles as well as the websites of the WHO and the CDC, a framework of roadmap was designed for how to use information technology tools in the vaccine coverage levels. Table 1 shows the purposes of using technology interventions, the type of tool and the final result of using tools in three phases including before, during and after vaccination processes.

### Approved concept models from two-stage Delphi

After reviewing valid sources in the field of technology interventions, as well as the websites of the WHO and the CDC, a framework of roadmap was designed for how to use information technology tools in the vaccine coverage levels. Four models were defined, which include the following:

1. Framework of E-health roadmap for application of IT tools in COVID-19 vaccination coverage
2. Vision, Mission, Goals and Values of IT tools for COVID-19 vaccine coverage
3. IT infrastructure framework in vaccine coverage
4. Stakeholders of vaccination coverage & E-health interventions

The conceptual models were presented and 14 experts in two Delphi stages provide opinions about models. Then, designed conceptual model send for 14 experts in the health information management fields; and they provide comments and opinions about it.

Actually, expert comment about the designed conceptual models was depicted in the Table 2.

**Table 2:** Expert Comments about conceptual models by Two Delphi Steps

Conceptual Models	Expert Comments
Framework of E-Health Roadmap for Application of IT tools in vaccine COVID-19 coverage	- Edit the first axis of 11 and then add the policy committee axis - Integration of all technologies used in three stages of vaccination - Provide explanations related to the dimensions of planning and organization - Add Portal as technology intervention - Determine international organizations
Vision, Mission, Goals and Values of IT tools for COVID-19 vaccine coverage	- Reengineering goals - Reengineering vision - Relationship management
IT infrastructure framework in vaccine coverage	-Add family level to existing levels -Add access component to existing levels -Add information governance component to national level
Stakeholders of vaccine coverage & E-Health interventions	- Separation of governmental and non-governmental organizations

### Framework of E-health roadmap for application of IT tools in COVID-19 vaccination coverage

**Fig. 2:**
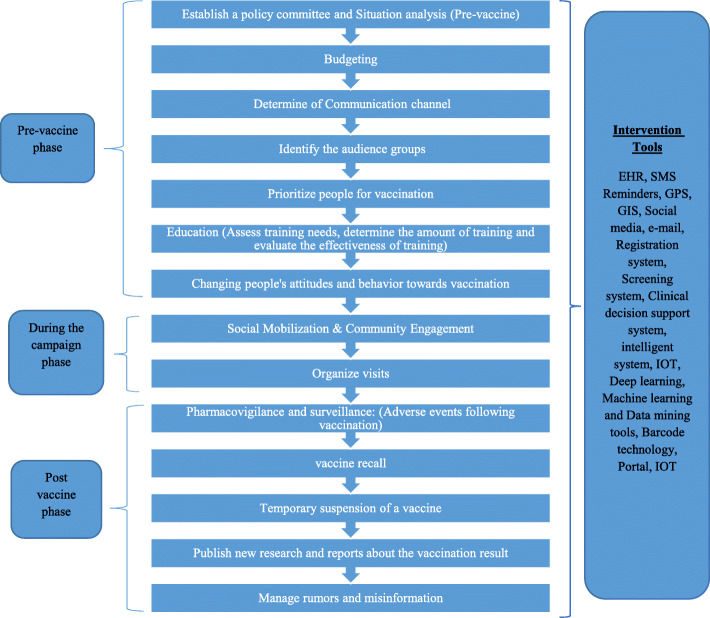
Framework of E-Health Roadmap for Application of IT tools in vaccine COVID-19 coverage

The proposed e-health road map not only assists government, health care providers, and payers for suitable use of information and communication technologies, it also improves improving quality, safety, and cost-effective processes associated with the delivery of health care. This roadmap has 14 axes in three phases including: Pre- vaccination, during the campaign phase and post vaccination phase as illustrated in Fig. 2.

#### Pre- vaccination phases

##### Establish a policy committee and situation analysis (pre-vaccination)

The tasks of the Policy and Planning Committee are: Develop a comprehensive economic management plan, managing clinical guidelines and policies, organizing the program information management system, management of educational programs, management of research and development related to the program, and identify and modify the system and methods of program implementation.

The analysis of the size and distribution of the health workforce based on its ability to provide the current health care package. A detailed situation analysis and classifying service delivery have been conducted via E-health technologies. At this stage, these seem necessary to prepare a national vaccination program, monitor this vaccination process and the steps of import and rapid clearance, ability to track vaccines, organize the required expenses and budgets, and identify the target population.

##### Changing people’s attitudes and behavior towards vaccination

Awareness among various stakeholders can increase by using different media. More ever HIT tools such as SMS and health portals can improve attitude of citizens and have potential benefits resulting from vaccine distributions.

##### Budgeting

Assess the affordability of vaccination programs and resource allocation are the key factors in budgeting of vaccination coverage. An effective and systematic budget process via IT innovations promotes accountability and establishes spending guidelines for governments.

##### Determine communication channel

It is necessary to aware about the primary information sources and safety communication channels. These communication channels can be media, news, and social media.

##### Identify the audience groups

The target population groups are the persons or groups or people identified according to clear goals of COVID-​19 vaccination programs.

##### Changing people’s attitudes and behaviors towards vaccination

Many factors can be effective in changing people’s attitudes. Efforts should be concentrated on motivating and strengthen positive attitudes toward vaccination by different information interventions. These innovations include virtual campaigns to raise risk perception and faster vaccination acceptance.

In this way, obstacles such as the stress from vaccine use must be removed.

##### Education (assess training needs, determine the amount of training and evaluate the effectiveness of training)

Training of COVID-19 vaccination can be done by the Health Center’s Welfare and Infectious Diseases Training Unit. In these education sessions, the necessary training including safe injection, vaccine side effects care and how to register vaccination services in the electronic system will be provided to target group by health workers and caregivers.

#### During the campaign phase

##### Social mobilization & community engagement

Organizing a vaccination mobilization requires good managerial power and technical knowledge. Responsibilities for each part of the program should be clearly defined and delegated to specific individuals or institutions, and national guidelines for emergency safety should be prepared as soon as possible.

##### Organize visits

Health care managers should organize visits to cover urban and rural vaccination areas.

#### Post vaccination phase

##### Pharmacovigilance and surveillance: (adverse events following vaccination)

The Department of Health should conduct a post-vaccination screening to check the any side effects for people who receiving the Coronavirus vaccine. It also possible through social media whether they had any symptoms such as swelling, fatigue and fever at least 37.5 degrees Celsius after the vaccine.

##### Vaccine recalls

Reminders can be sent to patients, careers, or the general population at the time of vaccination or overdue, depending on age or other risk factors by different ways including letter, postcard, phone call, or text message. Calls can send when vaccinations are overdue. Reminders are done for a variety of reasons including missed or missed appointments, unfamiliarity with vaccination schedules, and concerns about it. For reminders to be successful, vaccination records and contacts information must be accurate and up-to-date, reminders must be legible, and vaccination services must be available.

##### Temporary suspension of a vaccine

Communications should state the reasons for the suspensions, the decision-making process that addresses the uncertainties, and safety measures before restarting the vaccine program. Suspensions are generally reflecting cautious and reflect a cautious and safe approach to vaccines. Vaccine replacement is a planned procedure to improve the safety and effectiveness of the immunization program. Vaccines are often replaced with updated and modified products. If people are not aware of the reasons for vaccine replacement, they may be concerned and need to be sure of the purpose of the replacement.

##### New research findings related to vaccination results and publish reports on the vaccination process

Subsequently, research and development activities are replaced and the results are published.

##### Manage rumors and misinformation

Misinformation defined as an overabundance of information—some accurate and some not. Misinformation about COVID-19 vaccination has increased. Some solutions of information technology include: 1. direct and effective communication between scientists and the public via modern and traditional media, 2. improve websites and portals of public health organizations and vaccine centers via search engines, 3. advertise the accounts of public health personnel and pharmacists on popular social media platforms, 4. publish the posts of public health and medical professionals, 5. monitor social media platforms to control rumor messages, 6. share personal experiences on social media to combat misinformation, 7. awareness campaigns for vaccine coverage, 8. develop educational material and speed the share of evidence-based contents, 9. develop e-health communication strategy. (Fig. 2).

### Vision, Mission, goals and values of IT tools for COVID-19 vaccine coverage

**Fig. 3:**
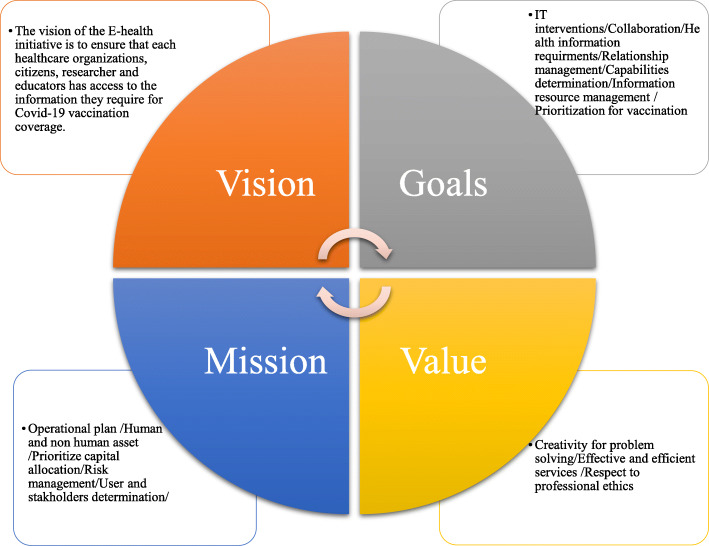
Vision, Mission, Goals and Values of IT tools for COVID-19 vaccine coverage

Vision, goals, mission, and values statement is an approach to help policy makers and mangers accomplish what it has set out to do and helps provide a framework for good vaccination coverage. The summary information is shown in Fig. 3.

#### Vision

The vision of the E-health initiative is to ensure that each health care organizations, citizens, researcher and educators has access to the information they require for COVID-19 vaccination coverage.

#### Goals

- Collaboration of health care organizations and Ministry of health are involved in COVID-19 vaccination coverage by E-health interventions. These organizations can share ideas and best practices in technology context.
- Responsibility to address information needs of the citizens, the health care providers, researchers and educators.
- Information resource management (IRM): IRM of vaccination coverage is a management process that refers to the identification, creation, capture, interpretation and distribution of information resource as a critical asset to support in devising policy and decision making.
- Prioritization for vaccination and clinical activity support: information technology can support clinical fields by three phases: Pre-vaccination phase, Post-vaccination phase and rehabilitation/improvement and death phase.

#### Mission

Use of E-health to facilitate data collection, processing, archiving and dissemination for COVID-19 vaccination coverage.

#### Values

- Creativity for problem solving by E-health
- Effective and efficient services by information technology
- Respect to professional ethics

### IT infrastructure framework in vaccination coverage

**Fig. 4:**
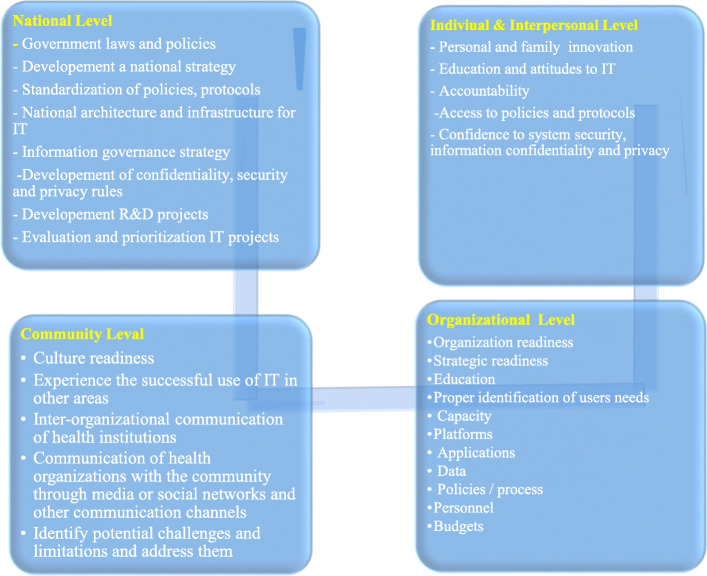
IT infrastructure framework in vaccine coverage

In this study, four levels were considered for the infrastructure framework. These levels include: national level, community level, individual & Interpersonal level and organizational level. 28 components were defined in these four levels as depicted in the Fig. 4.

### Stakeholders of vaccination coverage & E-health interventions

**Fig. 5:**
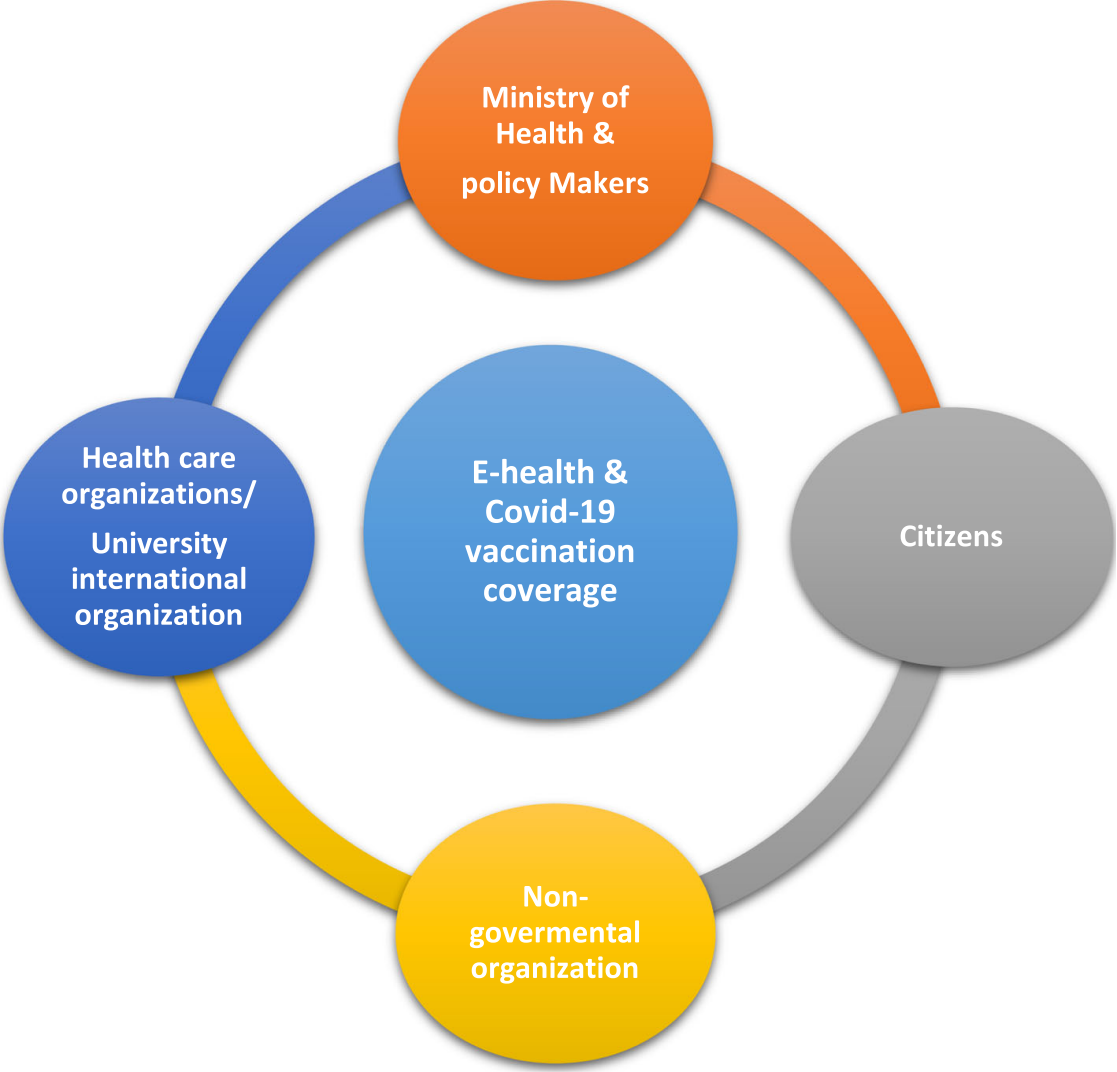
Stakeholders of vaccine coverage & E-health interventions

Figure 5 was illustrated stakeholders of vaccination coverage and E-health interventions. Ministry of Health, policy makers, citizens, nongovernmental organization, health care organizations, universities and international organization are considered as stakeholders.

## Discussion

The finalized roadmap for application of IT tools has 14 axes in three phases including: Pre- vaccination, during the campaign phase and post vaccination phase.

In this study, 10 articles related to vaccination coverage and E-health interventions were reviewed. After reviewing the valid articles, as well as WHO and CDC websites. E-health Roadmap for COVID-19 Vaccine Coverage were compiled in four sections and the model was finalized in the Delphi stage. In this study, 14 strategic axes of COVID-19 vaccination coverage were presented in three phases before, during and after vaccination. IT interventions in three phases were including: EHR, SMS reminders, GPS, GIS, social media, E-mail, registration system, screening system, clinical decision support system, intelligent system, portal, IOT, deep learning, machine learning and data mining tools, and barcode technology.

Integration of technologies facilitates the vaccination coverage process. GIS big data analyzing and IOT data can help managers and policy makers for prioritizing [23]. Spatial analysts can help vaccine distribution by gathering comprehensive information [36].

IT interventions should lead to the identification of best practices and clinical interventions. On the other hand responsibility in the use of information technology and attention to security principles is also important [37]. New technologies such as mobile applications, artificial intelligence, the Internet of Things and big data analytics are increasingly being used to combat epidemics and inequalities [38]. Big data analytics also facilitates vaccine development [39]. Online health portals and knowledge management systems can also be effective in data interactions, resource management, cost reduction and increase efficiency and effectiveness [40]. In this study, the role of information technology in each of the strategic axes of vaccination coverage in the context of the e-health roadmap was discussed.

Another application of information technology tools is the interaction of patients, health care providers and stakeholders in the management of COVID-19 disease. Because people, public and private organizations play an important role in combating COVID-19, it is essential to connect, coordinate and support them through emerging technologies [37].

A Sharing experiences and group decisions with the help of collaborative technologies plays an important role in the management of COVID-19 disease [41]. Therefore, one of the principles needs in communities is to invest in information technology infrastructures to deal with the impact of COVID-19 and public health crises [42]. Feasibility, readiness and flexibility of digital infrastructures are also important [43].

Another important issue is the change on human behavior and the effects of new technologies in this field. Information technology provides equitable access and online medical advice to the general public [37].

Non-governmental organizations(NGOs) can accelerate the introduction of new vaccines against COVID-19 disease [44]. NGOs are also considered in this study and they have a direct relationship with the general public.

It seems necessary to pay attention to the IT infrastructures at the national level during COVID-19. According to Wickramasinghe et al. framework for assessing E-health have the four main pre-requisites including the information technology infrastructure, the standardized policies, protocols and procedures, user access and accessibility policies and infrastructures, governmental regulations and roles as well as the impacts of IT education [45]. For example with proper health IT infrastructure can connect to smartphones and provide comprehensive information about people at risk. Existence of appropriate infrastructures enables the management of manpower, equipment and time [46]. In this study, infrastructure was presented in 4 levels. It is suggested that future studies should be done to address security and information confidentiality challenges.

## Conclusions

The technology-based roadmap is one plan in the form of a transfer strategy that aligns goals with specific technical solutions and helps to meet them. This roadmap empowers decision makers to decide on alternative paths and achieve goals.

The accurate data are beneficial for analyzing the pre-vaccination situations including budgeting, determine of communication channel and prioritizing people for vaccination and education. During the campaign, it applies to social mobilization & community engagement and organizing visits. IT-based interventions also use to monitor for probable adverse effects after vaccination, vaccine recall and suspension of the vaccine.

Planning is one of the important steps before IT project implementation. In this phase detailed planning of all parts that and creating a roadmap is crucial for easier and faster implementation process.

Given this framework, the existence of these four components will improve vaccination coverage. Access to these technologies and ongoing training for all stakeholders can pave the way for greater effectiveness of vaccination programs. Defects in any of these components reduce the effectiveness of E-health in this area and the expected results will not be achieved. The government is required to enact regulations to facilitate the exchange of health care information between stakeholders and protect the privacy and rights of users.

The roadmap clarifies the communication channel between planners, decision makers, customers, sponsors and stakeholders. This lead to reach a consensus on the set of technologies to achieve goals and create satisfaction. Also it provides information to make better decisions-making.

## Data Availability

The data that supported the findings of this study are available from the corresponding author on request.
